# Effect of childhood maltreatment and brain-derived neurotrophic factor on brain morphology

**DOI:** 10.1093/scan/nsw086

**Published:** 2016-07-12

**Authors:** Laura S. van Velzen, Lianne Schmaal, Rick Jansen, Yuri Milaneschi, Esther M. Opmeer, Bernet M. Elzinga, Nic J. A. van der Wee, Dick J. Veltman, Brenda W. J. H. Penninx

**Affiliations:** ^1^Department of Psychiatry and Neuroscience Campus Amsterdam, VU University Medical Center and GGZ inGeest, Amsterdam, the Netherlands; ^2^Department of Neuroscience, University of Groningen, NeuroImaging Center, University Medical Center Groningen, Groningen, the Netherlands; ^3^Institute of Psychology and Leiden Institute for Brain and Cognition (LIBC), Leiden University, Leiden, the Netherlands; ^4^Institute of Psychiatry and Leiden Institute for Brain and Cognition (LIBC), Leiden University, Leiden, the Netherlands,; ^5^Department of Psychiatry and the EMGO+ Institute for Health and Care Research, VU University Medical Center, Amsterdam, the Netherlands

**Keywords:** childhood maltreatment, brain-derived neurotrophic factor, BDNF, gene expression, brain structure

## Abstract

Childhood maltreatment (CM) has been associated with altered brain morphology, which may partly be due to a direct impact on neural growth, e.g. through the brain-derived neurotrophic factor (BDNF) pathway. Findings on CM, BDNF and brain volume are inconsistent and have never accounted for the entire BDNF pathway. We examined the effects of CM, BDNF (genotype, gene expression and protein level) and their interactions on hippocampus, amygdala and anterior cingulate cortex (ACC) morphology. Data were collected from patients with depression and/or an anxiety disorder and healthy subjects within the Netherlands Study of Depression and Anxiety (NESDA) (*N* = 289). CM was assessed using the Childhood Trauma Interview. BDNF Val66Met genotype, gene expression and serum protein levels were determined in blood and T1 MRI scans were acquired at 3T. Regional brain morphology was assessed using FreeSurfer. Covariate-adjusted linear regression analyses were performed. Amygdala volume was lower in maltreated individuals. This was more pronounced in maltreated met-allele carriers. The expected positive relationship between BDNF gene expression and volume of the amygdala is attenuated in maltreated subjects. Finally, decreased cortical thickness of the ACC was identified in maltreated subjects with the val/val genotype. CM was associated with altered brain morphology, partly in interaction with multiple levels of the BNDF pathway. Our results suggest that CM has different effects on brain morphology in met-carriers and val-homozygotes and that CM may disrupt the neuroprotective effect of BDNF.

## Introduction

Childhood maltreatment (CM), which comprises neglect and psychological, physical and sexual abuse, can lead to a wide range of adverse consequences. CM is associated with the development of various psychiatric disorders, including major depressive disorder, post-traumatic stress disorder and addiction (Molnar *et al.*, 2001; Spinhoven *et al.*, 2010), and has been associated with an unfavorable disease course and poor response to treatment (Hovens *et al.*, 2012; Nanni *et al.*, 2012). CM has also been associated with changes in regional brain morphology: decreased volume of the hippocampus (Dannlowski *et al.*, 2012; Teicher *et al.*, 2012; Hanson *et al.*, 2014), amygdala (Hanson *et al.*, 2014) and prefrontal cortex (Dannlowski *et al.*, 2012; Van Harmelen *et al.*, 2010) have been reported in individuals with a history of CM, although results regarding hippocampal and amygdala volume have been inconsistent (Van Harmelen *et al.*, 2010; Dannlowski *et al.*, 2012).

These inconsistent findings regarding the impact of CM on brain morphology could perhaps be explained by postulating that the effect of environmental stress is more prominent in individuals with a biological vulnerability. Previous studies have provided some evidence for an interaction effect of childhood maltreatment and a specific genotype of the brain-derived neurotrophic factor (BDNF) gene on regional brain volume. BDNF is a protein that is important for plasticity, neurogenesis and neuronal survival (Huang and Reichardt, 2001). The met-allele of the Val66Met Single-Nucleotide Polymorphism (SNP) of the BDNF gene, coding for a replacement of the amino acid valine (val) by methionine (met), has been associated with decreased activity-dependent secretion of BDNF (Egan *et al.*, 2003; Chen *et al.*, 2004). Previous studies investigating a BDNF gene by CM interaction have shown that Met-carriers of the BDNF gene with a history of CM have decreased volume of the anterior cingulate cortex (ACC) (Gerritsen *et al.*, 2012), hippocampus (Molendijk *et al.*, 2012; Carballedo *et al.*, 2013; Frodl *et al.*, 2014) and amygdala (Gatt *et al.*, 2009) compared to met-carriers without CM and individuals with a val/val genotype, although again results have been inconsistent (Gerritsen *et al.*, 2012).

The above mentioned studies focused on the Val66Met genotype. However, Val66Met genotype is only one element of the BDNF pathway. Previous studies have not examined other elements of the BDNF pathway including BDNF gene expression levels and serum protein levels. Therefore, in the current study, we examined the effect of CM and different markers in the BDNF pathway, and the interaction between CM and BDNF on volume of the hippocampus and amygdala and cortical thickness and surface area of the ACC. We expected an overall negative effect of CM on regional brain morphology. Consistent with previous studies, we hypothesized a gene-environment interaction effect showing a stronger decrease in amygdala and hippocampus volume and ACC thickness and surface area in met-carriers with a history of CM. Due to the important role of BDNF in neuronal survival, we expected a positive relationship between BDNF protein and gene expression levels and regional brain volume and cortical measures. To our knowledge, no previous studies have examined the relationship between BDNF gene expression or BDNF protein levels and brain morphology in relation to CM, but we expected that stress may obscure the protective effect of BDNF gene expression or serum levels on regional brain morphology in maltreated subjects.

## Materials and methods

### Subjects

The Netherlands Study of Depression and Anxiety (NESDA) is a longitudinal cohort study, which aims to examine the naturalistic course of depression and anxiety in a total of 2981 participants. NESDA includes subjects with depressive and/or anxiety disorder as well as subjects without a lifetime psychiatric diagnosis. Subjects were recruited from the community, general practitioners and specialized mental health care institutions (for details please see Penninx *et al.*, 2009).

A subgroup of NESDA patients and healthy controls were asked to participate in the NESDA neuroimaging study (*N* = 301). Inclusion criteria for the imaging study were a DSM-IV diagnosis of major depressive disorder (MDD) and/or anxiety disorder (social anxiety disorder and/or panic disorder and/or generalized anxiety disorder) in the six months preceding the interview for patients and no history of psychiatric disorders for controls. These diagnoses were established using the Composite International Diagnostic Interview (CIDI version 2.1) (Wittchen, 1994). Exclusion criteria for patients and controls were abuse or dependency of drugs or alcohol in the past year, general MRI contraindications and presence or history of a severe internal or neurological disorder. Additional exclusion criteria were use of psychotropic medication other than stable use of SSRIs or infrequent benzodiazepine use for patients and use of any psychoactive medication for healthy controls. The Ethical Review Boards of the three participating centers (i.e. Academic Medical Center Amsterdam, University Medical Center Groningen and Leiden University Medical Center) approved this study and all subjects provided written consent.

In the current study we included all healthy controls and patients with a 6-month diagnosis of MDD and/or an anxiety disorder. We excluded twelve participants due to poor image quality, leaving a total of 289 subjects in our study.

### Maltreatment

Childhood maltreatment was assessed with the Nemesis Childhood Trauma Interview (De Graaf *et al.*, 2002). In this semi-structured interview, participants were asked whether they had ever experienced emotional neglect, psychological abuse, physical abuse or sexual abuse before the age of 16 (see supplements for a detailed description). As these forms of abuse often occur together (Hovens *et al.*, 2010), it is difficult to examine effects related to specific forms of maltreatment. Therefore, we used a broad definition of CM and classified subjects as having a history of childhood abuse if they reported at least one type of maltreatment.

### Imaging

Imaging was performed on 3T Philips MR scanners (Philips, Best, The Netherlands) at the three participating centers (Leiden University Medical Center, Amsterdam Medical Center and University Medical Center Groningen). In Amsterdam, a SENSE-6 channel head coil was used, while the other sites used a SENSE-8 channel head coil. Anatomical scans were acquired using a sagittal three-dimensional gradient-echo T1-weighted sequence (TR: 9 ms; TE: 3.5 ms; matrix: 256_256; voxel size: 1 mm^3^; 170 slices)

Cortical reconstruction and volumetric segmentation were performed using FreeSurfer image analysis suite (version 5.3; Martinos Center for Biomedical Imaging, Harvard-MIT, Boston, MA; http://surfer.nmr.mgh.harvard.edu/). FreeSurfer includes motion correction and averaging, Talairach transformation, removal of non-brain tissue, segmentation of subcortical structures and cortical regions, intensity normalization and cortical reconstruction. For quality assessment, a visual inspection of all subcortical structures and cortical regions was performed, using a protocol developed by the ENIGMA consortium (http://enigma.ini.usc.edu/protocols/imaging-protocols/).

We chose volumes of the amygdala and hippocampus and cortical thickness and surface area of the rostral and caudal ACC as regions of interest in the current study, because of their specific association with both CM and BDNF (Gatt *et al.*, 2009; Gerritsen *et al.*, 2012; Frodl *et al.*, 2014).

### Brain-derived neurotrophic factor (BDNF)

#### Val66met genotype

Methods for biological sample collection and DNA extraction have been described previously (Boomsma *et al.*, 2008; Abdellaoui *et al.*, 2013). In brief, genotyping was performed on multiple chip platforms in different, and partially overlapping subsets of the total NESDA sample (Affymetrix-Perlegen 5.0 and Affymetrix 6.0). Quality checks and imputation methods have been detailed in a previous report and are described in the supplement (Nivard *et al.*, 2014). In subsequent analyses, we compared subjects who had at least one met-allele to val-val homozygotes (dominant model).

#### Gene expression measurement

RNA processing and measurements have likewise been outlined previously (Jansen *et al.*, 2014) and are described in the supplement. In this study, we calculated the mean BDNF expression across all five probe sets after correcting for technical covariates (i.e. plate number, position on plate, month and hour of blood withdrawal, hemoglobin level and days between blood withdrawal and RNA extraction).

#### Serum protein measurement

Blood withdrawal was performed in the morning between 7:30 and 9:30 after subjects had fasted overnight. Serum was extracted and stored at −85 °C. Protein levels were measured using the Emax ImmunoAssay system from Promega (for this procedure, see Bus *et al.*, 2011). All serum BDNF protein levels (expressed in nanograms per milliliter) were above the reliable detection limit of the ELISA kit (1.56 ng/ml).

### Statistical analyses

#### Sample characteristics

To examine differences in demographical variables, BDNF measures and brain structure between individuals who had and those who had not experienced CM, we used independent sample *t*-tests and χ^2^ tests. We considered all results significant if *P* < 05.

#### BDNF correlations

Partial correlation coefficients were calculated to examine the relationship between BDNF gene expression and BDNF serum levels, while correcting for age, sex and education level. Point-biserial correlation coefficients were calculated with similar covariates to investigate the association between BDNF Val66Met genotype and gene expression levels and between Val66Met genotype and serum protein levels.

#### Repeated measures ANOVA analyses

For our primary analyses, we performed repeated measures ANOVA analyses in SPSS 20 (IBM) to examine the main effects of CM on subcortical brain volume and cortical thickness. Furthermore, main effects of BDNF Val66Met (dummy coded 0 for met-carriers and 1 for val-allele homozygotes), gene expression levels and BDNF serum protein levels on brain morphology were explored. In addition, repeated measure ANOVA analyses were performed to examine the interaction of CM with Val66Met genotype, BDNF gene expression levels and serum protein levels on brain morphology in different models for each BDNF marker. Hemisphere (left or right) was added to the model as a within-group factor to investigate whether there were significant two-way (BDNF*hemisphere or CM*hemsiphere) or three-way (BDNF*CM*hemisphere) lateralization effects. If a significant interaction was observed, post-hoc tests were performed to examine by which hemisphere the results were driven.

Significant interactions were followed by two-sample *t*-tests (Bonferroni corrected for the number of post-hoc comparisons) or stratified regression analyses to examine group differences. In secondary analyses, potential confounding effects of SSRI use, smoking, alcohol use and population structure were controlled for by performing linear regression analyses with these variables as additional covariates (see below).

#### Covariates

Potential variance due to age, sex, education level (in years), scan site, presence of depression (coded as a dummy variable: yes/no), presence of an anxiety disorder (coded as a dummy variable: yes/no) and intracranial volume was corrected for all regression and ANOVA analyses. In secondary analyses we also corrected for smoking (coded as a dummy variable: current smoker *vs* non-smoker), alcohol use (number of alcohol drinks per week) and SSRI use (coded as a dummy variable: yes/no). Analyses focusing on Val66Met SNP were additionally adjusted for three ancestry-informative principal components derived from GWAS data (Abdellaoui *et al.*, 2013) to take possible population stratification into account.

### Effect of psychiatric diagnosis

As maltreatment, depression and anxiety disorders are strongly associated and we examine the effect of maltreatment in a sample that includes patients with depression and/or anxiety disorders, we performed additional analyses to examine if presence of a psychiatric disorder has a similar effect as CM on brain morphology. Presence of a psychiatric diagnosis (coded as a dummy: yes/no) was entered into repeated measure ANOVA analyses with age, sex, education, scan site and intracranial volume added as covariates.

## Results

### Sample characteristics

Table 1 shows the demographic and biological characteristics of our total sample (*N* = 289) and stratified for CM. Subjects with a history of CM (*N* = 146) had smaller amygdala volumes (*P *= 0.017) and more often had a diagnosis of depressive and/or anxiety disorders (both *P *< 0.01) than subjects who were not maltreated (*N* = 143). Age, sex, education level, total intracranial volume, Val66Met genotype, BDNF gene expression levels and serum protein levels did not differ between maltreated and non-maltreated subjects.

**Table 1.** Sample characteristics and differences between maltreated and non-maltreated subjects

Descriptive information	All subjects *N* = 289	Childhood maltreatment *N* = 146	No childhood maltreatment *N* = 143	*T*/*F*/*X*^2^ (df)	*P*-value	Cohen’s D
Demographic variables						
Age (years; s.d.)	37.69 (10.16)	38.43 (9.70)	36.92 (10.59)	*T* = −1.263 (287)	0.208	0.149
Sex (female/male)(% male)	32.9%	32.2%	32.9%	*X*^2^ = 0.000 (1)	1.00	0.000
Education (years; s.d.)	12.81 (3.20)	12.60 (3.29)	13.02 (3.09)	*T* = 1.113 (287)	0.267	−0.132
Scan site (Amsterdam/Leiden/Groningen)	94/107/88	49/53/44	45/54/44	*X*^2^ = 0.148 (2)	0.928	0.045
Brain morphology						
ICV (liter; s.d.)	1.5081 (0.183)	1.5079 (0.176)	1.5084 (0.190)	*F* = 0.060 (1)	0.807^e^	−0.002
Mean amygdala volume (mm^3^; s.d)	1637.34 (201.29)	1609.31 (209.46)	1665.97 (189.05)	*F* = 5.722 (1)	0.017^f^	−0.284
Mean hippocampal volume (mm^3^; s.d.)	3989.63 (423.48)	3965.69 (418.80)	4014.07 (428.29)	*F* = 0.849 (1)	0.358^f^	−0.114
Mean thickness rostral ACC (mm; s.d.)	2.57 (0.16)	2.55 (0.17)	2.59 (0.15)	*F* = 2.765 (1)	0.097^f^	−0.250
Mean thickness caudal ACC (mm; s.d.)	2.39 (0.18)	2.38 (0.17)	2.41 (0.18)	*F* = 0.924 (1)	0.337^f^	−0.171
Surface area rostral ACC (mm; s.d.)	805.60 (131.75)	808.24 (135.98)	802.92 (127.73)	*F* = 0.326 (1)	0.568^f^	0.040
Surface area caudal ACC (mm; s.d.)	755.92 (129.65)	748.47 (122.57)	763.48 (136.46)	*F* = 1.326 (1)	0.251^f^	−0.116
Brain-derived neurotrophic factor						
Genotype (% met carriers)^a^	33.6%	32.2%	35.0%	*X*^2^ = 0.400 (1)	0.527	0.074
Gene expression^b^	2.19 (0.12)	2.18 (0.11)	2.19 (0.13)	*F* = 0.799 (1)	0.373^g^	−0.083
Serum protein levels (ng/ml)^c^	8.99 (3.12)	9.08 (3.03)	8.90 (3.22)	*F* = 0.069 (1)	0.793^g^	0.058
Psychiatry and lifestyle						
Depression (%)^d^	54.0%	63.7%	44.1%	*X*^2^ = 11.221 (1)	0.001	0.402
Anxiety disorder (%)^d^	55.7%	65.1%	46.2%	*X*^2^ = 10.475 (1)	0.001	0.388
Smoking (% yes)	33.6%	33.2%	35.0%	*X*^2^ = 0.249 (1)	0.618	0.058
SSRI use (% yes)	27.3%	31.5%	23.1%	*X*^2^ = 2.585 (1)	0.108	0.190
Alcohol use (drinks per week)	5.14 (7.79)	4.63 (8.39)	5.68 (7.12)	*T* = 1.143 (286)	0.254	−0.135

Data are shown as mean (standard deviation) or as frequencies.

ACC: anterior cingulate cortex; ICV: intracranial volume; s.d.: standard deviation.

^a^Due to missing values genotype data was available for 255 subjects, of which 130 had a history of maltreatment.

^b^Due to missing values gene expression data was available for 195 subjects, of which 107 had a history of maltreatment.

^c^Due to missing values protein serum levels was available for 282 subjects, of which 143 had a history of maltreatment.

^d^Presence of an anxiety and/or depressive disorder.

^e^Corrected for age, sex, education level, scan site.

^f^Corrected for age, sex, education level, scan site, intracranial volume.

^g^Corrected for age, sex, education level.

Due to missing BDNF values, 255 subjects were included in Val66Met genotype analyses, 195 subjects in BDNF gene expression analyses and 282 subjects in BDNF protein analyses.

### Childhood maltreatment and brain morphology

In covariate adjusted repeated measures ANOVA analyses, presence of CM was associated with decreased volume of the amygdala (*P *= 0.038) (Table 2). CM was unrelated to hippocampal volume or ACC thickness and surface area. There was no significant interaction between CM and hemisphere on brain morphology (Table S1), indicating that findings were not driven by one hemisphere.

**Table 2.** Main effects and interaction effects of childhood maltreatment and BDNF on brain morphology measures

Regions of interest	Childhood maltreatment *N* = 289				BDNF genotype *N* = 255				Childhood maltreatment x genotype interaction *N* = 255			
	Df	*F*	*P*-value	Partial η^2^	Df	*F*	p-value	Partial η^2^	Df	F	*P*-value	Partial η^2^
Hippocampus	1,279	0.330	0.566	0.001	1,242	0.018	0.894	0.000	1,240	1.908	0.168	0.008
Amygdala	1,279	4.334	0.038	0.015	1,242	0.124	0.725	0.001	1,240	21.588	0.000	0.083
Th. caudal ACC	1,279	0.091	0.763	0.000	1,242	0.264	0.608	0.001	1,240	7.315	0.007	0.030
Th. rostral ACC	1,279	0.894	0.345	0.003	1,242	0.762	0.384	0.003	1,240	4.822	0.029	0.020
SA caudal ACC	1,278	0.317	0.574	0.001	1,241	3.578	0.060	0.015	1,240	1.033	0.310	0.004
SA rostral ACC	1,278	1.010	0.316	0.004	1,241	2.209	0.138	0.009	1,240	0.190	0.663	0.001
					BDNF gene expression N = 195				Childhood maltreatment x expression interaction *N* = 195			
					Df	*F*	p-value	Partial η^2^	Df	F	p-value	Partial η^2^
Hippocampus					1,185	0.990	0.321	0.005	1,183	0.649	0.422	0.004
Amygdala					1,185	3.062	0.082	0.016	1,183	6.838	0.010	0.036
Th, caudal ACC					1,185	0.211	0.646	0.001	1,183	0.679	0.411	0.004
Th. rostral ACC					1,185	0.018	0.894	0.000	1,183	4.826	0.029	0.026
SA caudal ACC					1,184	0.379	0.539	0.002	1,182	0.908	0.342	0.005
SA rostral ACC					1,184	0.373	0.542	0.002	1,182	0.052	0.819	0.000
					BDNF protein levels N = 282				Childhood maltreatment x protein interaction N = 282			
					Df	*F*	p-value	Partial η^2^	Df	F	p-value	Partial η^2^
Hippocampus					1,272	1.715	0.191	0.006	1,270	0.811	0.368	0.003
Amygdala					1,272	0.080	0.777	0.000	1,270	0.390	0.533	0.001
Th. caudal ACC					1,272	0.036	0.849	0.000	1,270	1.097	0.296	0.004
Th. rostral ACC					1,272	0.043	0.835	0.000	1,270	0.052	0.819	0.000
SA caudal ACC					1,271	0.226	0.635	0.001	1,269	2.345	0.127	0.009
SA rostral ACC					1,271	0.755	0.386	0.003	1,269	1.818	0.179	0.007

Results of repeated measure ANOVA analyses. The dependent variables (hippocampal and amygdala volume, cortical thickness and surface area of the ACC) are shown in the first column. Presented results are corrected for differences in age, sex, educational level, diagnosis of depression and diagnosis of anxiety, scan site and intracranial volume. ACC: anterior cingulate cortex; SA: surface area Th.:cortical thickness.

### BDNF and brain morphology

BDNF Val66Met, gene expression levels or serum protein levels were not associated with volume of the amygdala and hippocampus or ACC morphology (Table 2). However, at a trend level, there was an association between BDNF Val66Met genotype and surface area of the caudal ACC (*P *= 0.060), with increased surface area observed in met-carriers (M:773.81; SD:123.44) compared to individuals with a val/val genotype (M:742.91; SD:133.45). There was also a trend for a positive association between BDNF gene expression levels and amygdala volume (*P *= 0.082). The above mentioned findings were not driven by one hemisphere as there were no significant BDNF*hemisphere interaction effects (Table S1).

### Interaction between maltreatment and BDNF

#### Interaction between maltreatment and Val66Met

**Fig. 1.**
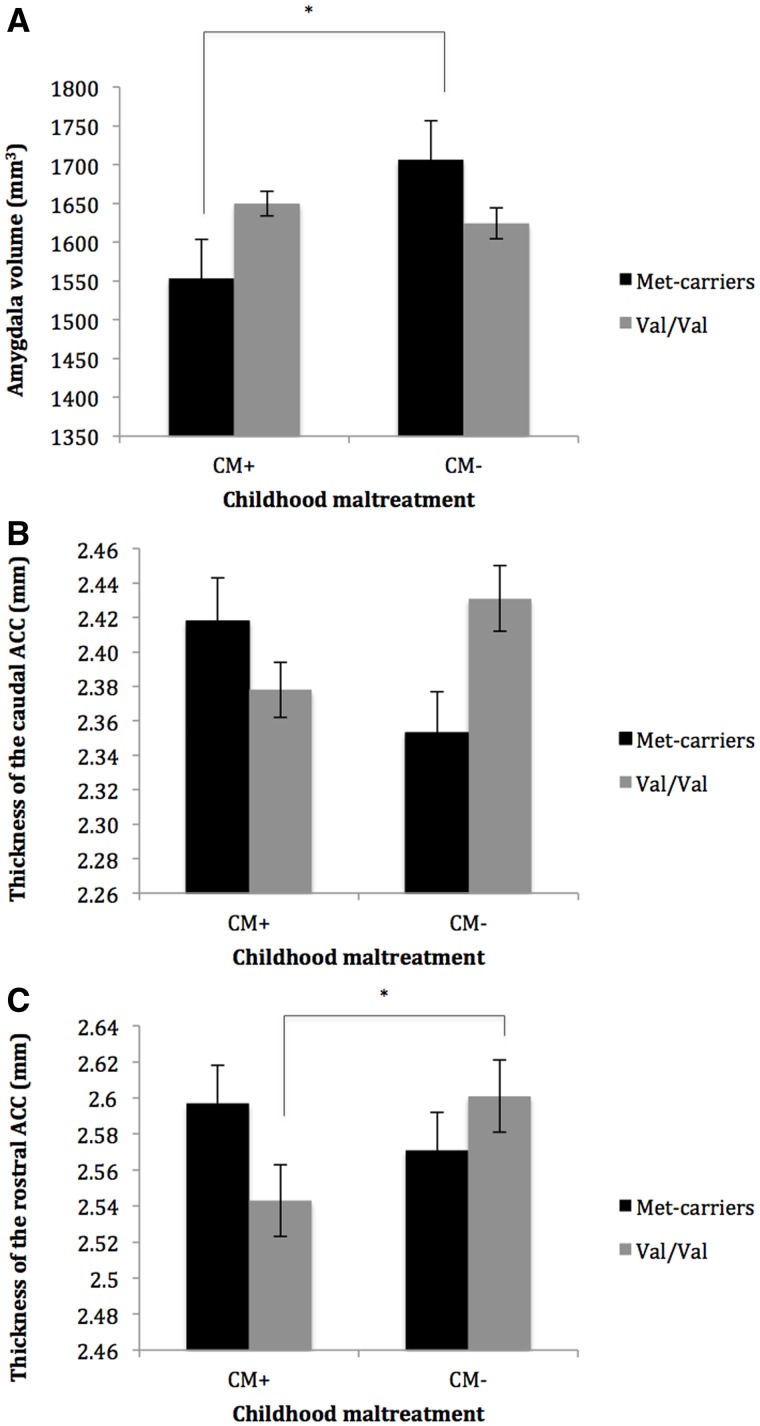
BDNF Val66Met genotype and brain morphology in maltreated and non-maltreated subjects. (A) An interaction effect between Val66Met and childhood maltreatment on amygdala volume. Post-hoc *t*-tests were performed. *:significant at *P* < 0.05 (Bonferroni-corrected). (B) Interaction effect between Val66Met and childhood maltreatment on thickness of the caudal ACC. Post-hoc tests do not show significant differences between groups. (C) An interaction effect between Val66Met and childhood maltreatment on thickness of the rostral anterior cingulate cortex. Post-hoc *t*-tests were performed *: significant at *P*<0.05 (Bonferroni-corrected). All presented results are corrected for differences in age, gender, educational level, scan site and intracranial volume. Error bars indicate the standard error of the mean. ACC: anterior cingulate cortex; CM-: no history of childhood maltreatment; CM+: history of childhood maltreatment.

We observed an interaction effect between CM and Val66Met genotype on amygdala volume (*P *< 0.001; Table 2 and Figure 1). Post-hoc ANCOVA tests indicated that in individuals with a history of CM, the met-allele was associated with decreased amygdala volume (M: 1555.23; SE: 25.00), compared to non-maltreated met-carriers (M: 1704.94; SE: 24.94) (*P *= 0.003, Bonferroni-corrected). There was no significant CM*Val66Met*hemisphere interaction effect (*P *= 0.099; Table S1), suggesting that this finding was not driven by one hemisphere.

We also observed an interaction effect between CM and Val66Met on thickness of the caudal and rostral ACC (Figure 1; *P *= 0.007 and *P *= 0.029, respectively). Post-hoc tests did not show significant differences between groups in caudal ACC thickness, but rostral ACC thickness was lower in maltreated individuals with a val/val genotype (M: 2.547; SE:0.013), compared to non-maltreated individuals with a val/val genotype (M:2.593; SE:0.017) (*P *= 0.051, Bonferroni-corrected). This CM*Val66Met interaction effect appeared to be driven by the right hemisphere ACC (post-hoc regression analysis: standardized beta: −0.322; SE: 0.119; *T*=−2.707, *P *= 0.007) and not the left hemisphere ACC (standardized beta: −0.062; SE: 0.113 *T*=−0.547, *P *= 0.585), as there was a significant three-way interaction between CM, Val66Met genotype and hemisphere on rostral ACC thickness (Table S1).

Hippocampal volume and ACC surface area were not associated with an interaction between CM and Val66Met genotype (Table 2).

#### Interaction between maltreatment and BDNF gene expression

**Fig. 2.**
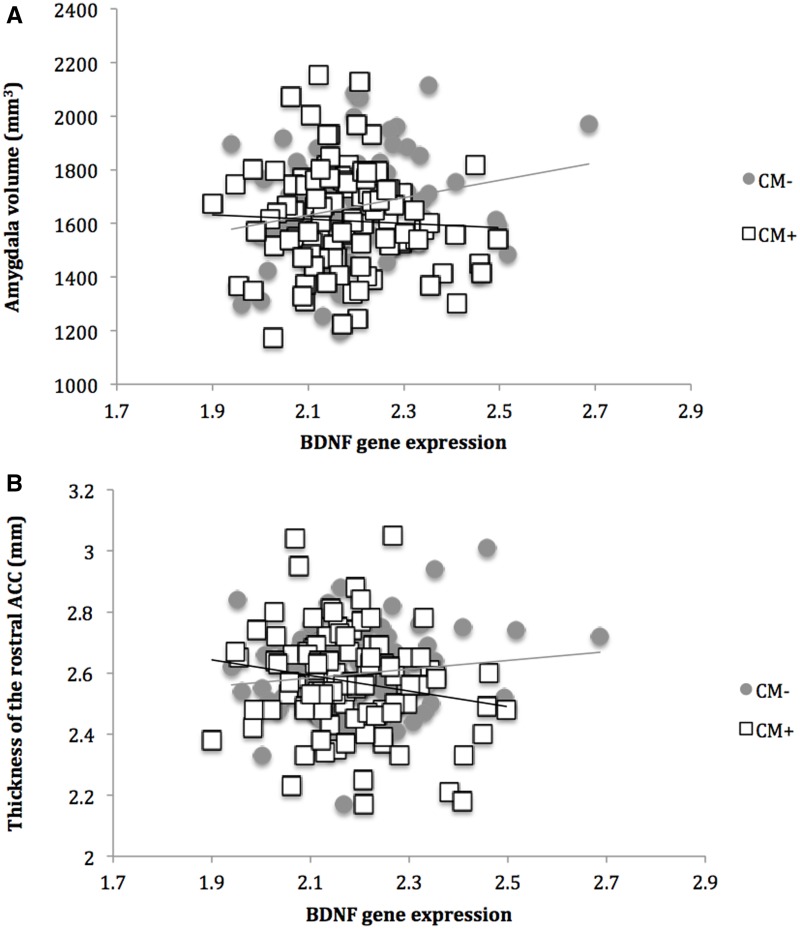
BDNF gene expression and brain morphology in maltreated and non-maltreated subjects. Shown here are results of a stratified linear regression analysis. (A) Interaction effect between BDNF gene expression and childhood maltreatment on amygdala volume. There is a positive relationship between BDNF gene expression and volume of the amygdala in subjects without a history of maltreatment, which is absent in maltreated subjects. (B) Interaction effect between BDNF gene expression and childhood maltreatment on rostral ACC thickness. Stratified analyses reveal a positive, but non-significant relationship in non-maltreated subjects and a negative, but non-significant relationship in maltreated subjects. CM−: no history of childhood maltreatment; CM+: history of childhood maltreatment.

A significant interaction effect between CM and BDNF gene expression levels on amygdala volume was observed (*P *= 0.010; Table 2 and Figure 2). Post-hoc regression analyses stratified for CM revealed that BDNF gene expression was positively associated with amygdala volume in individuals without CM (standardized beta: 0.252 SE: 0.094; *T* = 2.687; *P *= 0.009), while this relationship was absent in individuals with a history of CM (standardized beta: -0.066; SE: 0.091;*T*=−0.722; *P *= 0.472).

There was also a significant interaction between BDNF gene expression and CM on rostral ACC thickness (*P *= 0.029; Table 2 and Figure 2). Stratified post-hoc analysis revealed a trendwise positive relationship between gene expression and thickness in individuals without CM (standardized beta: 0.190; SE: 0.102; *T* = 1.869; *P *= 0.065) and a negative, but non-significant relationship in individuals with a history of CM (standardized beta: −0.125; SE: 0.096; *T* =−1.297; *P *= 0.198).

Hippocampal volume and caudal ACC thickness were unrelated to an interaction between CM and BDNF expression. In addition, no significant CM*gene expression*hemisphere interaction effects were observed (Table S1).

#### Interaction between maltreatment and BDNF serum protein levels

We did not observe an interaction effect between BDNF protein levels and CM on amygdala and hippocampus volumes or ACC cortical thickness and surface area (Table 2).

### Psychiatric diagnosis, BDNF and brain volume

While depression and anxiety disorders were corrected for in all analyses, we performed additional analyses to examine to what extent our findings could also be explained by psychiatric status. Presence of a psychiatric disorder was associated with decreased volume of the hippocampus, rostral ACC thickness and caudal ACC surface area (*P *= 0.029, *P* < 0.001 and *P = *0.008, respectively; Table 3) and amygdala volume and caudal ACC thickness at trend-level (*P *= 0.064 and *P *= 0.059, respectively, Table 3). We also observed an interaction between psychiatric diagnosis and Val66Met genotype on thickness of the rostral ACC and surface area of the caudal ACC (*P *= 0.051 and 0.007; Table 3). Post-hoc ANCOVA analyses did not reveal significant differences in rostral ACC thickness between groups, but did reveal a trend towards decreased caudal ACC surface area in patients with val/val genotype (M 738.62; SD: 128.53) compared to healthy met-carriers (M: 819.95; SD: 141.79) (*P *= 0.058; Bonferroni-corrected). These results suggest that the CM*BDNF genotype effect on the ACC may be partly explained by a psychiatry*BDNF interaction effect.

**Table 3.** Main effects and interaction effects of psychiatric diagnosis and BDNF on brain morphology measures

Regions of interest	Psychiatric diagnosis *N* = 289					Psychiatric diagnosis x genotype interaction *N* = 255		
	Df	*F*	*P*-value	Partial η^2^	Df	*F*	*P*-value	Partial η^2^
Hippocampus	1,281	4.807	0.029	0.017	1,242	0.305	0.581	0.001
Amygdala	1,281	3.453	0.064	0.012	1,242	1.166	0.281	0.005
Thickness caudal ACC	1,281	3.600	0.059	0.013	1,242	0.272	0.603	0.001
Thickness rostral ACC	1,281	14.559	0.000	0.049	1,242	3.852	0.051	0.016
SA caudal ACC	1,280	7.042	0.008	0.025	1,241	7.503	0.007	0.030
SA rostral ACC	1,280	1.995	0.159	0.007	1,241	0.222	0.638	0.001
						Psychiatric diagnosis x expression interaction *N* = 195		
					Df	*F*	*P*-value	Partial η^2^
Hippocampus					1,185	0.017	0.895	0.000
Amygdala					1,185	1.071	0.302	0.006
Thickness caudal ACC					1,185	2.164	0.143	0.012
Thickness rostral ACC					1,185	17.049	0.000	0.084
SA caudal ACC					1,184	0.892	0.346	0.005
SA rostral ACC					1,184	0.093	0.761	0.001
						Psychiatric diagnosis x protein interaction *N* = 282		
					Df	F	*P*-value	Partial η^2^
Hippocampus					1,272	0.735	0.392	0.003
Amygdala					1,272	0.003	0.954	0.000
Thickness caudal ACC					1,272	0.008	0.927	0.000
Thickness rostral ACC					1,272	0.249	0.618	0.001
SA caudal ACC					1,271	0.277	0.599	0.001
SA rostral ACC					1,271	0.285	0.594	0.001

Results of repeated measure ANOVA analyses. The dependent variables (hippocampal and amygdala volume, cortical thickness and surface area of the ACC) are shown in the first column. Presented results are corrected for differences in age, sex, educational level, scan site and intracranial volume. ACC: anterior cingulate cortex; SA: surface area.

Furthermore, there was an interaction effect between psychiatric diagnosis and BDNF gene expression levels on thickness of the rostral ACC (*P* < 0.001; Table 3). Stratified analyses showed that there was a positive effect of BDNF gene expression on rostral ACC thickness in healthy controls (standardized beta: 0.510; SE: 0.172; *T* =2.972; *P *= 0.006), which was absent in patients (standardized beta: −0.141; SE: 0.075; *T* =−1.888; *P*= 0.061).

There was no interaction effect of psychiatric diagnosis and BDNF on amygdala volume, suggesting that the observed interaction effects of CM, Val66Met genotype and BDNF gene expression levels on amygdala volume are independent of psychiatric status.

### Secondary analyses

#### Correction for additional confounding variables

Results of the secondary analyses correcting for potential confounders (i.e. SSRI use, smoking and alcohol use) are presented in table S2. Following this additional correction, we still observed a main effect of CM on amygdala volume (*P *= 0.030). The interaction effects of CM and Val66Met on amygdala volume and thickness of the caudal and rostral ACC remained significant (*P *< 0.001, *P *= 0.007 and *P *= 0.022, respectively). Furthermore, the interaction effects between CM and BDNF gene expression on amygdala volume and rostral ACC thickness also remained significant (*P *= 0.005 and *P *= 0.033).

#### Correlation between Val66Met, BDNF gene expression and amygdala volume

Since we observed both a significant interaction effect between CM*Val66Met and between CM* BDNF gene expression levels on amygdala volume, we additionally examined whether these two interaction effects related to each other. To this aim we performed an additional post-hoc linear regression analysis in which we included these variables, covariates, as well as both interaction terms with amygdala volume as outcome. Both interaction terms remained significant (CM*gene expression: standardized beta=−0.228; SE = 0.084; *T*=−2.703; *P *= 0.008; CM*gene: standardized beta = 0.431; SE =0.121; *T* = 3.571; *P *< 0.001), indicating that the observed interaction effects were independent from each other. This independence between BDNF genotype and gene expression is further demonstrated by a lack of association between the different BDNF markers. The correlation between BDNF gene expression levels and protein levels was not significant (*R*=−0.041, *P *= 0.582). There was no significant correlation between Val66Met genotype and gene expression levels (*R*_pb _= 0.05; *P *= 0.5) and between Val66Met genotype and protein levels. (*R*_pb _= 0.01; *P *= 0.869).

## Discussion

The aim of this study was to examine the effect of CM, BDNF and their interaction on volume of the amygdala and hippocampus and thickness of the ACC. This is the first study to examine multiple levels of the BDNF pathway—including the Val66Met SNP, gene expression and protein levels—in relation to CM. Amygdala volume was lower in subjects with a history of CM. Furthermore, we found a gene-environment interaction on amygdala volume and thickness of the ACC. We also observed these interaction effects at the gene expression level.

Overall, amygdala volume was lower in subjects who were exposed to CM. The amygdala is a key structure for processing emotional information including memory formation and has been implicated in many maltreatment-related disorders, including post-traumatic stress disorder and other anxiety disorders (Etkin and Wager, 2007). Previous studies have reported conflicting results, including increased (Pechtel *et al.*, 2014), decreased (Edmiston *et al.*, 2011; Hanson *et al.*, 2014) and unaltered amygdala volume (Woon and Hedges, 2008) in maltreated subjects. Rodent studies have shown an increase in dendritic arborisation in the amygdala in response to stress (Vyas *et al.*, 2002; Padival *et al.*, 2013), which may result in an increase in volume. It has been proposed that CM may cause an initial increase in amygdala volume and activity, which over time may be followed by neurodegeneration and reduced volume of the amygdala in maltreated subjects (Hanson *et al.*, 2014). In support of this hypothesis, prolonged stress was associated with degeneration of amygdala cells in adult rats (Ding *et al.*, 2010). Our finding of lower amygdala volume in maltreated adults also fits this model, although longitudinal research is needed to corroborate our results. Given the role of the amygdala in emotion processing, we speculate that decreased amygdala volume may underlie emotion regulation impairments in maltreated subjects (Dvir *et al.*, 2014; O’Mahen *et al.*, 2015).

We did not find evidence for a direct effect of BDNF on brain morphology. As BDNF is expressed in the prefrontal cortex, amygdala and hippocampus (Conner *et al.*, 1997; Dwivedi *et al.*, 2003), and is crucial for neurogenesis and neuronal survival (Huang and Reichardt, 2001), we expected a positive relationship between BDNF (gene expression levels and serum protein levels) and measures of regional brain morphology. Previous work has shown a positive relationship between serum protein levels, hippocampal volume and memory performance (Erickson *et al.*, 2010), however this finding was not replicated in later studies (Driscoll *et al.*, 2012; Kim *et al.*, 2015). In the current study, the evidence for an effect of BDNF gene expression or serum protein levels on brain morphology was limited. The effect of BDNF on brain volume may be small or BDNF levels may vary over time, preventing us from detecting an effect of current BDNF levels on volume of the amygdala, hippocampus and ACC (Piccinni *et al.*, 2008). We also did not see an effect of BDNF genotype on brain volume and therefore were unable to replicate initial findings of decreased hippocampus volume in carriers of the met-allele (Bueller *et al.*, 2006; M L Molendijk *et al.*, 2012), however recent meta-analyses suggest this effect is non-existent and is related to publication bias (Marc L Molendijk *et al.*, 2012; Harrisberger *et al.*, 2014).

Results of our interaction analyses suggest that smaller amygdala volumes in individuals with a history of CM are even more prominent in carriers of the met-allele of the BDNF gene. The met-allele may increase vulnerability to CM related morphological changes in the amygdala due to decreased activity-dependent secretion of BDNF (Egan *et al.*, 2003) or increased HPA-axis reactivity to stress (Colzato *et al.*, 2011; Yu *et al.*, 2012) in met-carriers. The amygdala has many glucocorticoid receptors (Wang *et al.*, 2013) and high glucocorticoid levels have been associated with decreased amygdala volume (Schuhmacher *et al.*, 2012). Therefore increased glucocorticoid levels in response to stress during development may result in decreased amygdala volume in met carriers. A history of maltreatment and the presence of a met-allele appear to interact to lead to more pronounced reduced volume in maltreated met-carriers. We did not find evidence for a similar interaction between depression and/or anxiety and Val66Met genotype on amygdala volume, suggesting that the interaction effects on amygdala volume may not be related to depression and/or anxiety. It is important to note that this is a cross-sectional study and that longitudinal studies are needed to address any association between maltreatment—related changes in brain morphology and development of maltreatment related psychiatric disorders.

Although BDNF gene expression levels were not significantly lower in maltreated subjects, alterations in the interaction between BDNF gene expression and CM on volume of the amygdala were observed. In line of what would be expected, higher levels of gene expression were associated with greater amygdala volume and ACC thickness in non-maltreated individuals, but this effect was absent in maltreated subjects. This lack of an association between BDNF gene expression levels and amygdala volume could be suggestive of an absence of a neuroprotective effect of BDNF in maltreated subjects. This disruption could in turn be explained by disrupted BDNF signalling downstream of the receptor. For instance, pro-inflammatory cytokines and glucocorticoids have been shown to interact and affect BDNF signaling (see review by Numakawa *et al.*, 2014). It has been reported that BDNF-stimulated signaling in the AKT and ERK pathways and BDNF-related facilitation of long term potentiation (LTP) in the hippocampus is reduced after administration of pro-inflammatory cytokine interleukin 1β (Tong *et al.*, 2008; Tong *et al.*, 2012). Glucocorticoids have also been found to suppress BDNF signaling in the MAPK/ERK pathway (Kumamaru *et al.*, 2011). Increased levels of pro-inflammatory cytokines and dysregulation of the HPA-axis have been observed in maltreated individuals (Gonzalez, 2013; Coelho *et al.*, 2014). High levels of glucocorticoids and pro-inflammatory cytokines may thus interfere with the effects downstream of the BDNF receptor in maltreated individuals thereby interfering with the neuroprotective effect of BDNF, yielding an absence of a positive association between BDNF gene expression levels and amygdala volume.

Our results suggest that the observed CM-Val66Met genotype and CM-BDNF gene expression interaction effects on amygdala morphology are independent, indicating that the effect of the Val66Met gene on amygdala volume is not explained by BDNF gene expression effects on amygdala volume in maltreated participants. This is in line with our observation of a lack of an correlation between the different markers. This is also in accordance with previous studies, which did not find a difference in plasma BDNF levels between met-carriers and val/val homozygotes (Terracciano *et al.*, 2013; Chen *et al.*, 2015). Perhaps a one-to-one mapping of genotype to gene expression and serum protein effects is also not to be expected because BDNF serum protein and gene expression levels are influenced by multiple genes, epigenetic effects and other biological markers, including cytokines, glucocorticoids and sex hormones (Carbone and Handa, 2013; Terracciano *et al.*, 2013; Calabrese *et al.*, 2014), which may obscure the direct associations between markers of BDNF.

The CM*BDNF interaction effects we observed are complex, as maltreatment was related to decreased amygdala volume in met-carriers and reduced cortical thickness of the right rostral ACC in val-homozygotes. The rostral anterior cingulate is important for emotion regulation and is highly interconnected with the amygdala (Etkin *et al.*, 2011). Stress has been associated with decreased dendritic arborisation in the anterior cingulate cortex (Radley *et al.*, 2004; Liston *et al.*, 2006), which may decrease cortical thickness. Reduced ACC volume and cortical thickness have previously been reported in maltreated subjects (Cohen *et al.*, 2006; Treadway *et al.*, 2009; Gerritsen *et al.*, 2012; Kelly *et al.*, 2013), but results of our study suggest that this effect may have been driven by subjects with a val/val genotype. As a similar interaction effect between the presence of a psychiatric diagnosis and BDNF was found on rostral ACC thickness, our findings may be driven by or related to depression and/or anxiety disorders. It remains unclear why the effect of CM on cortical thickness of the ACC was specifically observed in val-allele homozygotes, as to our knowledge decreased BDNF activity-dependent secretion and structural deficits have only been found in met-carriers (e.g. Egan *et al.*, 2003; Pezawas *et al.*, 2004; Montag *et al.*, 2009). More research is needed to replicate this effect and examine possible mechanisms.

In contrast to some, but not all, previous studies we did not find evidence for structural changes in the hippocampus of maltreated subjects or a relationship between BDNF and hippocampal volume. Although a CM*BDNF gene expression*hemisphere interaction effect was observed (Table S1), post-hoc tests did not reveal an effect of BDNF gene expression on the left or right hippocampus in maltreated or non-maltreated subjects (data not shown). Furthermore, we did not find a main effect of maltreatment on hippocampal volume. Several manual segmentation studies have shown decreased hippocampal volume in individuals with a history of CM (Bremner *et al.*, 1997; Weniger *et al.*, 2009)(with sample sizes of *N* = 34 and *N* = 42, respectively), voxel-based morphometry studies in larger samples were not able to replicate this finding (Van Harmelen *et al.*, 2010; Gerritsen *et al.*, 2012) (*N* = 568 and *N* = 181), or only at trend-level (Cohen *et al.*, 2006) (*N* = 256), suggesting that the earlier results may have been false positive results or may have been related to differences in analysis technique (e.g. manual segmentation, FreeSurfer segmentation or voxel-based morphometry) (Frodl and O’Keane, 2013).

One of the strengths of the present study is that Val66Met genotype, BDNF gene expression and protein serum levels were determined in a large study sample, providing adequate power to control for various potentially confounding variables. An obvious limitation is that gene expression and protein levels were determined in peripheral blood. However, preclinical studies have shown that peripheral serum protein levels reflect cortical and hippocampal BDNF (Karege *et al.*, 2002; Sullivan *et al.*, 2006; Klein *et al.*, 2011). A second limitation is that BDNF gene expression and Val66Met genotype information was not available for every subject. Analyses revealed that subjects that could not be included in the BDNF gene expression analyses, more often had a diagnosis of depression and/or anxiety than individuals that were not included (data not shown). Third, this study was performed in a heterogeneous sample, consisting of healthy controls and patients with major depression and/or an anxiety disorders, however we corrected for the presence of depression and/or anxiety in all CM analyses and have also examined the effect of depression and/or anxiety (in interaction with BDNF) on brain morphology to examine to what extent our findings could also be explained by psychiatric status.

In conclusion, CM is associated with lower amygdala volume, a region that has been implicated in many maltreatment-related psychiatric disorders. Decreased amygdala volume may underlie emotion regulation impairments. This finding of smaller amygdala volume in maltreated individuals appears to be even more pronounced in individuals carrying a met-allele, while ACC thickness was specifically decreased in maltreated val-homozygotes. The observed interaction effects are thus complex, as childhood maltreatment has different effects on brain morphology in met-carriers and val-homozoygotes. Furthermore, the individual components of the BDNF pathway did not show identical relationships with brain morphology. These findings add to our understanding of the effect of early life stress on the brain by showing that maltreatment-related changes in brain structure are related to BDNF genotype and gene expression, but not protein level. Further research is needed to elucidate on potential causal mechanisms between BDNF, CM and their effects on brain morphology, also in relation to vulnerability of developing a maltreatment-related psychiatric disorder.

## Supplementary data

Supplementary data are available at *SCAN* online.

Supplementary Data

## References

[nsw086-B1] Abdellaoui A. Hottenga J.J. de Knijff P., (2013). Population structure, migration, and diversifying selection in the Netherlands. European Journal of Human Genetics: EJHG, 21(11), 1277–85. 23531865 10.1038/ejhg.2013.48 PMC3798851

[nsw086-B2] Boomsma D.I. Willemsen G. Sullivan P.F., (2008). Genome-wide association of major depression: description of samples for the GAIN major depressive disorder study: NTR and NESDA biobank projects. European Journal of Human Genetics: EJHG, 16(3), 335–42. 18197199 10.1038/sj.ejhg.5201979

[nsw086-B3] Bremner J.D. Randall P. Vermetten E., (1997). Magnetic resonance imaging-based measurement of hippocampal volume in posttraumatic stress disorder related to childhood physical and sexual abuse—a preliminary report. Biological Psychiatry, 41(1), 23–32. 8988792 10.1016/s0006-3223(96)00162-x PMC3229101

[nsw086-B4] Bueller J.A. Aftab M. Sen S. Gomez-Hassan D. Burmeister M. Zubieta J.K. (2006). BDNF Val66Met allele is associated with reduced hippocampal volume in healthy subjects. Biological Psychiatry, 59(9), 812–5. 16442082 10.1016/j.biopsych.2005.09.022

[nsw086-B5] Bus B.A. Molendijk M.L. Penninx B.J.W.H., (2011). Determinants of serum brain-derived neurotrophic factor. Psychoneuroendocrinology, 36(2),228–39. 20702043 10.1016/j.psyneuen.2010.07.013

[nsw086-B6] Calabrese F. Rossetti A.C. Racagni G. Gass P. Riva M.A. Molteni R. (2014). Brain-derived neurotrophic factor: a bridge between inflammation and neuroplasticity. Frontiers in Cellular Neuroscience, 8, 1–7. 25565964 10.3389/fncel.2014.00430 PMC4273623

[nsw086-B7] Carballedo A. Morris D. Zill P., (2013). Brain-derived neurotrophic factor Val66Met polymorphism and early life adversity affect hippocampal volume. American Journal of Medical Genetics, Part B: Neuropsychiatric Genetics, 162, 183–90. 10.1002/ajmg.b.32130 23341118

[nsw086-B8] Carbone D.L. Handa R.J. (2013). Sex and stress hormone influences on the expression and activity of brain-derived neurotrophic factor. Neuroscience, 239, 295–303. 23211562 10.1016/j.neuroscience.2012.10.073 PMC3609934

[nsw086-B9] Chen S.L. Lee S.Y. Chang Y.H., (2015). The BDNF Val66Met polymorphism and plasma brain-derived neurotrophic factor levels in Han Chinese heroin-dependent patients. Scientific Reports, 5, 8148. 25640280 10.1038/srep08148 PMC4313085

[nsw086-B10] Chen Z.Y. Patel P.D. Sant G., (2004). Variant brain-derived neurotrophic factor (BDNF) (Met66) alters the intracellular trafficking and activity-dependent secretion of wild-type BDNF in neurosecretory cells and cortical neurons. The Journal of Neuroscience: The Official Journal of the Society for Neuroscience, 24(18), 4401–11. 15128854 10.1523/JNEUROSCI.0348-04.2004 PMC6729450

[nsw086-B11] Coelho R. Viola T.W. Walss-Bass C. Brietzke E. Grassi-Oliveira R. (2014). Childhood maltreatment and inflammatory markers: a systematic review. Acta Psychiatrica Scandinavica, 129, 180–92. 24205846 10.1111/acps.12217

[nsw086-B12] Cohen R.A. Grieve S. Hoth K.F., (2006). Early life stress and morphometry of the adult anterior cingulate cortex and caudate nuclei. Biological Psychiatry, 59(10), 975–82. 16616722 10.1016/j.biopsych.2005.12.016

[nsw086-B13] Colzato L.S. Van der Does A.J.W. Kouwenhoven C. Elzinga B.M. Hommel B. (2011). BDNF Val66Met polymorphism is associated with higher anticipatory cortisol stress response, anxiety, and alcohol consumption in healthy adults. Psychoneuroendocrinology, 36(10), 1562–9. 21596481 10.1016/j.psyneuen.2011.04.010

[nsw086-B14] Conner J.M. Lauterborn J.C. Yan Q. Gall C.M. Varon S. (1997). Distribution of brain-derived neurotrophic factor (BDNF) protein and mRNA in the normal adult rat CNS: evidence for anterograde axonal transport. The Journal of Neuroscience: The Official Journal of the Society for Neuroscience, 17(7), 2295–313. 9065491 10.1523/JNEUROSCI.17-07-02295.1997 PMC6573520

[nsw086-B15] Dannlowski U. Stuhrmann A. Beutelmann V., (2012). Limbic scars: long-term consequences of childhood maltreatment revealed by functional and structural magnetic resonance imaging. Biological Psychiatry, 71(4), 286–93. 22112927 10.1016/j.biopsych.2011.10.021

[nsw086-B16] De Graaf R. Bijl R.V. Smit F. Vollebergh W.A.M. Spijker J. (2002). Risk factors for 12-month comorbidity of mood, anxiety, and substance use disorders: findings from the Netherlands mental health survey and incidence study. American Journal of Psychiatry, 159, 620–9. 11925301 10.1176/appi.ajp.159.4.620

[nsw086-B17] Ding J. Han F. Shi Y. (2010). Single-prolonged stress induces apoptosis in the amygdala in a rat model of post-traumatic stress disorder. Journal of Psychiatric Research, 44(1), 48–55. 19586638 10.1016/j.jpsychires.2009.06.001

[nsw086-B18] Driscoll I. Martin B. An Y., (2012). Plasma BDNF is associated with age-related white matter atrophy but not with cognitive function in older, non-demented adults. PloS One, 7(4), e35217. 22523577 10.1371/journal.pone.0035217 PMC3327651

[nsw086-B19] Dvir Y. Ford J.D. Hill M. Frazier J.A. (2014). Childhood maltreatment, emotional dysregulation, and psychiatric comorbidities. Harvard Review of Psychiatry, 22(3), 149–61. 24704784 10.1097/HRP.0000000000000014 PMC4091823

[nsw086-B20] Dwivedi Y. Rizavi H.S. Conley R.R. Roberts R.C. Tamminga C.A. Pandey G.N. (2003). Altered gene expression of brain-derived neurotrophic factor and receptor tyrosine kinase B in postmortem brain of suicide subjects. Archives of General Psychiatry, 60(8), 804–15. 12912764 10.1001/archpsyc.60.8.804

[nsw086-B21] Edmiston E.E. Wang F. Mazure C.M., (2011). Corticostriatal-limbic gray matter morphology in adolescents with self-reported exposure to childhood maltreatment. Archives of Pediatrics and Adolescent Medicine, 165(12), 1069–77. 22147775 10.1001/archpediatrics.2011.565 PMC3607102

[nsw086-B22] Egan M.F. Kojima M. Callicott J.H., (2003). The BDNF val66met polymorphism affects activity-dependent secretion of BDNF and human memory and hippocampal function. Cell, 112, 257–69. 12553913 10.1016/s0092-8674(03)00035-7

[nsw086-B23] Erickson K.I. Prakash R.S. Voss M.W., (2010). BDNF is associated with age-related decline in Hippocampal volume. Journal of Neuroscience, 30(15), 5368–75. 20392958 10.1523/JNEUROSCI.6251-09.2010 PMC3069644

[nsw086-B24] Etkin A. Egner T. Kalisch R. (2011). Emotional processing in anterior cingulate and medial prefrontal cortex. Trends in Cognitive Sciences. 15(2), 85–93. 21167765 10.1016/j.tics.2010.11.004 PMC3035157

[nsw086-B25] Etkin A. Wager T.D. (2007). Functional neuroimaging of anxiety: a meta-analysis of emotional processing in PTSD, social anxiety disorder, and specific phobia. The American Journal of Psychiatry, 164(10), 1476–88. 17898336 10.1176/appi.ajp.2007.07030504 PMC3318959

[nsw086-B26] Frodl T. O’Keane V. (2013). How does the brain deal with cumulative stress? A review with focus on developmental stress, HPA axis function and hippocampal structure in humans. Neurobiology of Disease, 52, 24–37. 22426398 10.1016/j.nbd.2012.03.012

[nsw086-B27] Frodl T. Skokauskas N. Frey E.M. Morris D. Gill M. Carballedo A. (2014). BDNF Val66Met genotype interacts with childhood adversity and influences the formation of hippocampal subfields. Human Brain Mapping, 5783, 5776–83. 10.1002/hbm.22584 PMC6869047 25044977

[nsw086-B28] Gatt J.M. Nemeroff C.B. Dobson-Stone C., (2009). Interactions between BDNF Val66Met polymorphism and early life stress predict brain and arousal pathways to syndromal depression and anxiety. Molecular Psychiatry, 14(7), 681–95. 19153574 10.1038/mp.2008.143

[nsw086-B29] Gerritsen L. Tendolkar I. Franke B., (2012). BDNF Val66Met genotype modulates the effect of childhood adversity on subgenual anterior cingulate cortex volume in healthy subjects. Molecular Psychiatry, 17, 597–603. 21577214 10.1038/mp.2011.51

[nsw086-B30] Gonzalez A. (2013). The impact of childhood maltreatment on biological systems: implications for clinical interventions. Les Conséquences De La Maltraitance Pendant L’enfance Sur Les Systèmes Biologiques: Les Répercussions Sur Les Interventions Cliniques, 18(8), 415–8. PMC3887079 24426793

[nsw086-B31] Hanson J.L. Nacewicz B.M. Sutterer M.J., (2014). Behavioral problems after early life stress: contributions of the Hippocampus and amygdala. Biological Psychiatry, 77(4), 1–9. 10.1016/j.biopsych.2014.04.020 PMC4241384 24993057

[nsw086-B32] Harrisberger F. Spalek K. Smieskova R., (2014). The association of the BDNF Val66Met polymorphism and the hippocampal volumes in healthy humans: a joint meta-analysis of published and new data. Neuroscience and Biobehavioral Reviews, 42, 267–78. 24674929 10.1016/j.neubiorev.2014.03.011

[nsw086-B33] Hovens J.G.F.M. Giltay E.J. Wiersma J.E. Spinhoven P. Penninx B.W.J.H. Zitman F.G. (2012). Impact of childhood life events and trauma on the course of depressive and anxiety disorders. Acta Psychiatrica Scandinavica, 126, 198–207. 22268708 10.1111/j.1600-0447.2011.01828.x

[nsw086-B34] Hovens J.G.F.M. Wiersma J.E. Giltay E.J., (2010). Childhood life events and childhood trauma in adult patients with depressive, anxiety and comorbid disorders vs. controls. Acta Psychiatrica Scandinavica, 122(1), 66–74. 19878136 10.1111/j.1600-0447.2009.01491.x

[nsw086-B35] Huang E.J. Reichardt L.F. (2001). Neurotrophins: roles in neuronal development and function. Annual Review of Neuroscience, 24, 677–736. 10.1146/annurev.neuro.24.1.677 PMC2758233 11520916

[nsw086-B36] Jansen R. Batista S. Brooks A.I., (2014). Sex differences in the human peripheral blood transcriptome. BMC Genomics, 15, 33. 24438232 10.1186/1471-2164-15-33 PMC3904696

[nsw086-B37] Karege F. Schwald M. Cisse M. (2002). Postnatal developmental profile of brain-derived neurotrophic factor in rat brain and platelets. Neuroscience Letters, 328, 261–4. 12147321 10.1016/s0304-3940(02)00529-3

[nsw086-B38] Kelly P.A Viding E. Wallace G.L., (2013). Cortical thickness, surface area, and gyrification abnormalities in children exposed to maltreatment: neural markers of vulnerability?. Biological Psychiatry, 74(11), 845–52. 23954109 10.1016/j.biopsych.2013.06.020

[nsw086-B39] Kim A. Fagan A.M. Goate A.M. Benzinger T.L.S. Morris J.C. Head D. (2015). Lack of an association of BDNF Val66Met polymorphism and plasma BDNF with hippocampal volume and memory. Cognitive, Affective and Behavioral Neuroscience, 15(3), 625–43. 10.3758/s13415-015-0343-x PMC4529376 25784293

[nsw086-B40] Klein A.B. Williamson R. Santini M.A., (2011). Blood BDNF concentrations reflect brain-tissue BDNF levels across species. The International Journal of Neuropsychopharmacology/Official Scientific Journal of the Collegium Internationale Neuropsychopharmacologicum (CINP), 14, 347–53. 10.1017/S1461145710000738 20604989

[nsw086-B41] Kumamaru E. Numakawa T. Adachi N. Kunugi H. (2011). Glucocorticoid suppresses BDNF-stimulated MAPK/ERK pathway via inhibiting interaction of Shp2 with TrkB. FEBS Letters 585(20), 3224–8. 21946312 10.1016/j.febslet.2011.09.010

[nsw086-B42] Liston C. Miller M.M. Goldwater D.S., (2006). Stress-induced alterations in prefrontal cortical dendritic morphology predict selective impairments in perceptual attentional set-shifting. The Journal of Neuroscience: The Official Journal of the Society for Neuroscience, 26(30), 7870–4. 16870732 10.1523/JNEUROSCI.1184-06.2006 PMC6674229

[nsw086-B43] Molendijk M.L. Bus B.A. Spinhoven P., (2012). A systematic review and meta-analysis on the association between BDNF val 66 met and Hippocampal volume — a genuine effect or a winners curse? Neuropsychiatric Genetics, 159(6), 731–40. 10.1002/ajmg.b.32078 22815222

[nsw086-B44] Molendijk M.L. van Tol M.J. Penninx B.W.J.H., (2012). BDNF val66met affects hippocampal volume and emotion-related hippocampal memory activity. Translational Psychiatry, 2, e74. 22832736 10.1038/tp.2011.72 PMC3309548

[nsw086-B45] Molnar B.E. Buka S.L. Kessler R.C. (2001). Child sexual abuse and subsequent psychopathology: results from the national comorbidity survey. American Journal of Public Health, 91(5), 753–60. 11344883 10.2105/ajph.91.5.753 PMC1446666

[nsw086-B46] Montag C. Weber B. Fliessbach K. Elger C. Reuter M. (2009). The BDNF Val66Met polymorphism impacts parahippocampal and amygdala volume in healthy humans: incremental support for a genetic risk factor for depression. Psychological Medicine, 39, 1831–9. 19335934 10.1017/S0033291709005509

[nsw086-B47] Nanni V. Uher R. Danese A. (2012). Childhood maltreatment predicts unfavorable course of illness and treatment outcome in depression: a meta-analysis. American Journal of Psychiatry, 169, 141–51. 22420036 10.1176/appi.ajp.2011.11020335

[nsw086-B48] Nivard M.G. Mbarek H. Hottenga J.J., (2014). Further confirmation of the association between anxiety and CTNND2: Replication in humans. Genes, Brain and Behavior, 13, 195–201. 10.1111/gbb.12095 24256404

[nsw086-B49] Numakawa T. Richards M. Nakajima S., (2014). The role of brain-derived neurotrophic factor in comorbid depression: possible linkage with steroid hormones, cytokines, and nutrition. Frontiers in Psychiatry, 5, 136. 25309465 10.3389/fpsyt.2014.00136 PMC4175905

[nsw086-B50] O’Mahen H.A. Karl A. Moberly N. Fedock G. (2015). The association between childhood maltreatment and emotion regulation: two different mechanisms contributing to depression? Journal of Affective Disorders, 174, 287–95. 25528000 10.1016/j.jad.2014.11.028

[nsw086-B51] Padival M.A. Blume S.R. Rosenkranz J.A. (2013). Repeated restraint stress exerts different impact on structure of neurons in the lateral and basal nuclei of the amygdala. Neuroscience, 246, 230–42. 23660193 10.1016/j.neuroscience.2013.04.061 PMC3722557

[nsw086-B52] Pechtel P. Lyons-Ruth K. Anderson C.M. Teicher M.H. (2014). Sensitive periods of amygdala development: the role of maltreatment in preadolescence. NeuroImage, 97, 236–44. 24736182 10.1016/j.neuroimage.2014.04.025 PMC4258391

[nsw086-B53] Penninx B.J.W.H. Beekman A.T.F. Smit J.H., (2009). The Netherlands study of depression and anxiety (NESDA): rationale, objectives and methods. International Journal of Methods in Psychiatric Research, 17(3), 121–40. 10.1002/mpr.256 PMC6878352 18763692

[nsw086-B54] Pezawas L. Verchinski B.A. Mattay V.S., (2004). The brain-derived neurotrophic factor val66met polymorphism and variation in human cortical morphology. *The* Journal of Neuroscience: The Official Journal of the Society for Neuroscience, 24(45), 10099–102. 10.1523/JNEUROSCI.2680-04.2004 PMC6730170 15537879

[nsw086-B55] Piccinni A. Marazziti D. Del Debbio A., (2008). Diurnal variation of plasma brain-derived neurotrophic factor (BDNF) in humans: an analysis of sex differences. Chronobiology International 25(5), 819–26. 18780207 10.1080/07420520802387773

[nsw086-B56] Radley J.J. Sisti H.M. Hao J., (2004). Chronic behavioral stress induces apical dendritic reorganization in pyramidal neurons of the medial prefrontal cortex. Neuroscience 125(1), 1–6. 15051139 10.1016/j.neuroscience.2004.01.006

[nsw086-B57] Schuhmacher A. Mössner R. Jessen F., (2012). Association of amygdala volumes with cortisol secretion in unipolar depressed patients. Psychiatry Research – Neuroimaging, 202(2), 96–103. 22698761 10.1016/j.pscychresns.2011.09.007

[nsw086-B58] Spinhoven P. Elzinga B.M. Hovens J.G.F.M., (2010). The specificity of childhood adversities and negative life events across the life span to anxiety and depressive disorders. Journal of Affective Disorders, 126, 103–12. 20304501 10.1016/j.jad.2010.02.132

[nsw086-B59] Sullivan P.F. Fan C. Perou C.M. (2006). Evaluating the comparability of gene expression in blood and brain. American Journal of Medical Genetics. Part B, Neuropsychiatric Genetics: The Official Publication of the International Society of Psychiatric Genetics, 141B(3), 261–8. 10.1002/ajmg.b.30272 16526044

[nsw086-B60] Teicher M.H. Anderson C.M. Polcari A. (2012). PNAS Plus: childhood maltreatment is associated with reduced volume in the hippocampal subfields CA3, dentate gyrus, and subiculum. Proceedings of the National Academy of Sciences U S A, 109(35), E563–72. 10.1073/pnas.1115396109 PMC3295326 22331913

[nsw086-B61] Terracciano A. Piras M.G. Lobina M., (2013). Genetics of serum BDNF: meta-analysis of the Val66Met and genome-wide association study. World Journal of Biological Psychiatry ,14(8), 583–9. 22047184 10.3109/15622975.2011.616533 PMC3288597

[nsw086-B62] Tong L. Balazs R. Soiampornkul R. Thangnipon W. Cotman C.W. (2008). Interleukin-1 beta impairs brain derived neurotrophic factor-induced signal transduction. Neurobiology of Aging, 29(9), 1380–93. 17467122 10.1016/j.neurobiolaging.2007.02.027 PMC4052889

[nsw086-B63] Tong L. Prieto G.A. Kramár E.A., (2012). Brain-derived neurotrophic factor-dependent synaptic plasticity is suppressed by interleukin-1β via p38 mitogen-activated protein kinase. The Journal of Neuroscience: The Official Journal of the Society for Neuroscience, 32(49), 17714–24. 23223292 10.1523/JNEUROSCI.1253-12.2012 PMC3687587

[nsw086-B64] Treadway M.T. Grant M.M. Ding Z. Hollon S.D. Gore J.C. Shelton R.C. (2009). Early adverse events, HPA activity and rostral anterior cingulate volume in MDD. PloS One, 4(3), e4887. 19325704 10.1371/journal.pone.0004887 PMC2656617

[nsw086-B65] Van Harmelen A.L. Van Tol M.J. Van Der Wee N. J., (2010). Reduced medial prefrontal cortex volume in adults reporting childhood emotional maltreatment. Biological Psychiatry, 68(9), 832–8. 20692648 10.1016/j.biopsych.2010.06.011

[nsw086-B66] Vyas A. Mitra R. Shankaranarayana Rao B.S. Chattarji S. (2002). Chronic stress induces contrasting patterns of dendritic remodeling in hippocampal and amygdaloid neurons. The Journal of Neuroscience: The Official Journal of the Society for Neuroscience, 22(15), 6810–8. 12151561 10.1523/JNEUROSCI.22-15-06810.2002 PMC6758130

[nsw086-B67] Wang Q. Verweij E.W.E. Krugers H.J. Joels M. Swaab D.F. Lucassen P.J. (2013). Distribution of the glucocorticoid receptor in the human amygdala; changes in mood disorder patients. Brain Structure and Function, 219(5), 1615–26. 23748930 10.1007/s00429-013-0589-4

[nsw086-B68] Weniger G. Lange C. Sachsse U. Irle E. (2009). Reduced amygdala and hippocampus size in trauma-exposed women with borderline personality disorder and without posttraumatic stress disorder. Journal of Psychiatry and Neuroscience: JPN, 34(5), 383–8. 19721849 PMC2732745

[nsw086-B69] Wittchen H.U. (1994). Reliability and validity studies of the who–composite international diagnostic interview (CIDI): a critical review. Journal of Psychiatric Research, 28(I), 57–84. 8064641 10.1016/0022-3956(94)90036-1

[nsw086-B70] Woon F.L. Hedges D.W. (2008). Hippocampal and amygdala volumes in children and adults with childhood maltreatment-related posttraumatic stress disorder: a meta-analysis. Hippocampus, 18, 729–36. 18446827 10.1002/hipo.20437

[nsw086-B71] Yu H. Wang D.D. Wang Y. Liu T. Lee F.S. Chen Z.Y. (2012). Variant brain-derived neurotrophic factor val66met polymorphism alters vulnerability to stress and response to antidepressants. Journal of Neuroscience, 32(12), 4092–101. 22442074 10.1523/JNEUROSCI.5048-11.2012 PMC3319323

