# Supplementary material for: Effect of childhood maltreatment and brain-derived neurotrophic factor on brain morphology
Source: Soc Cogn Affect Neurosci. 2016 Jul 12;11(11):1841–52. doi: 10.1093/scan/nsw086 (PMC5091678; doi:10.1093/scan/nsw086)
Supplement: Supplementary Data [file supp_11_11_1841__index.html]

Effect of childhood maltreatment and brain-derived neurotrophic factor on brain morphology — Effect of childhood maltreatment and brain-derived neurotrophic factor on brain morphology — Supplementary Data 

# Effect of childhood maltreatment and brain-derived neurotrophic factor on brain morphology

## Supplementary Data

files

- Supplementary Data - docx file
