# Supplementary material for: Effect of childhood maltreatment and brain-derived neurotrophic factor on brain morphology
Source: Soc Cogn Affect Neurosci. 2016 Jul 12;11(11):1841–52. doi: 10.1093/scan/nsw086 (PMC5091678; doi:10.1093/scan/nsw086)
Supplement: Supplementary Data [file supp_nsw086_scan-15-537-File004.docx]

**Supplemental data**

Childhood trauma interview

In the childhood trauma interview, participants were asked the following questions: (1) Were you emotionally neglected, meaning nobody ever listened to you at home, your problems and experiences were ignored and you felt that there was no attention or support from your parents? (2) Were you psychologically abused, meaning being yelled at, falsely punished, subordinated to your siblings or being blackmailed? (3) Were you being abused physically, meaning being hit, kicked, beaten up or other types of physical abuse? (4) Were you sexually abused, meaning being touched or having to touch someone in a sexual way against your will? (5) Were you sexually abused, meaning being touched or having to touch someone in a sexual way against your will after the age of 16?

Val66Met genotype quality checks and imputation

Quality control was performed within and between chip platforms. Basic quality control steps for subjects included checks for European ancestry, sex inconsistencies, mendelian errors, missing rates and high genome-wide homozygosity. Genotype data were further checked based on Hardy-Weinberg equilibrium, minor allele frequencies (MAFs) and call rates. Data were imputed using the 1000 Genomes phase 1 INTEGRATED RELEASE version 3 ALL panel (28).The Val66Met BDNF SNP (rs6265) was typed in some of the panels used for imputation and all post-imputation QC criteria were met. R^2^ value was 0.99, indicating an almost perfect correlation between the imputed genotype and the true underlying genotype. The minor allele frequency was 0.21.

BDNF gene expression measurement

Venous blood samples were drawn in the morning after an overnight fast. Heparinized whole blood samples were transferred within 20 minutes of sampling into PAXgene Blood RNA tubes (Qiagen) and stored at −20°C. Samples were hybridized to Affymetrix U219 arrays (Affymetrix, Santa Clara, CA) containing 530,467 probes summarized in 49,293 probe sets. Array hybridization, washing, staining, and scanning were carried out in an Affymetrix GeneTitan System per the manufacturer’s protocol. Gene expression data were required to pass standard Affymetrix QC metrics (Affymetrix expression console) before further analysis. We excluded from further analysis probes that did not map uniquely to the hg19 (Genome Reference Consortium Human Build 37) reference genome sequence, as well as probes targeting a messenger RNA (mRNA) molecule resulting from transcription of a DNA sequence containing a single nucleotide polymorphism (based on the dbSNP137 common database). Normalized probe set expression values were obtained using Robust Multi‑array Average (RMA) normalization as implemented in the Affymetrix Power Tools software (APT, version 1.12.0, Affymetrix).

|  | Childhood maltreatment x hemisphere  N = 289 | | | | BDNF genotype x hemisphere  N = 255 | | | | Childhood maltreatment x genotype x hemisphere interaction N = 255 | | | |
| --- | --- | --- | --- | --- | --- | --- | --- | --- | --- | --- | --- | --- |
|  | Df | F | p-value | Partial η^2^ | Df | F | p-value | Partial η^2^ | Df | F | p-value | Partial η^2^ |
| Hippocampus | 1,279 | 0.501 | 0.480 | 0.002 | 1,242 | 0.584 | 0.445 | 0.002 | 1.241 | 0.004 | 0.953 | 0.000 |
| Amygdala | 1,279 | 1.785 | 0.183 | 0.006 | 1,242 | 1.617 | 0.205 | 0.007 | 1,241 | 2.749 | 0.099 | 0.011 |
| Thickness caudal ACC | 1,279 | 1.066 | 0.303 | 0.004 | 1,242 | 0.151 | 0.698 | 0.001 | 1,241 | 0.039 | 0.843 | 0.000 |
| Thickness rostral ACC | 1,279 | 1.565 | 0.212 | 0.006 | 1,242 | 0.142 | 0.707 | 0.001 | 1,241 | 4.631 | 0.032 | 0.019 |
| SA caudal ACC | 1,278 | 0.601 | 0.439 | 0.002 | 1,241 | 0.019 | 0.892 | 0.000 | 1,240 | 0.221 | 0.639 | 0.001 |
| SA rostral ACC | 1,278 | 0.201 | 0.654 | 0.001 | 1,241 | 0.772 | 0.380 | 0.003 | 1,240 | 4.783 | 0.030 | 0.020 |
|  | | | | | BDNF gene expression x hemisphere  N = 195 | | | | Childhood maltreatment x expression interaction x hemisphere N = 195 | | | |
|  | | | | | Df | F | p-value | Partial η^2^ | Df | F | p-value | Partial η^2^ |
| Hippocampus | | | | | 1,185 | 0.047 | 0.828 | 0.000 | 1,183 | 4.151 | 0.043 | 0.022 |
| Amygdala | | | | | 1,185 | 0.182 | 0.670 | 0.001 | 1,183 | 0.526 | 0.469 | 0.003 |
| Thickness caudal ACC | | | | | 1,185 | 0.009 | 0.925 | 0.000 | 1,183 | 0.042 | 0.839 | 0.000 |
| Thickness rostral ACC | | | | | 1,185 | 1.431 | 0.233 | 0.008 | 1,183 | 0.408 | 0.524 | 0.002 |
| SA caudal ACC | | | | | 1,184 | 0.257 | 0.613 | 0.001 | 1,182 | 0.056 | 0.813 | 0.000 |
| SA rostral ACC | | | | | 1,184 | 2.753 | 0.099 | 0.015 | 1,182 | 0.380 | 0.538 | 0.002 |
|  | | | | | BDNF protein levels x hemisphere  N = 282 | | | | Childhood maltreatment x protein interaction x hemisphere N = 282 | | | |
|  | | | | | Df | F | p-value | Partial η^2^ | Df | F | p-value | Partial η^2^ |
| Hippocampus | | | | | 1,272 | 0.301 | 0.584 | 0.001 | 1,270 | 1.411 | 0.236 | 0.005 |
| Amygdala | | | | | 1,272 | 1.313 | 0.253 | 0.005 | 1,270 | 3.199 | 0.075 | 0.012 |
| Thickness caudal ACC | | | | | 1,272 | 4.236 | 0.041 | 0.015 | 1,270 | 0.035 | 0.852 | 0.000 |
| Thickness rostral ACC | | | | | 1,272 | 3.251 | 0.072 | 0.012 | 1,270 | 0.016 | 0.900 | 0.000 |
| SA caudal ACC | | | | | 1,271 | 1.036 | 0.310 | 0.004 | 1,269 | 0.455 | 0.505 | 0.002 |
| SA rostral ACC | | | | | 1,271 | 0.694 | 0.406 | 0.003 | 1,269 | 0.000 | 0.991 | 0.000 |

Table S1. Results of repeated measures ANOVA analyses: interaction with hemisphere

|  | Childhood maltreatment  N = 289 | | | | BDNF genotype  N = 255 | | | | | Childhood maltreatment x genotype interaction N = 255 | | | |
| --- | --- | --- | --- | --- | --- | --- | --- | --- | --- | --- | --- | --- | --- |
|  | df | F | p-value | Partial η^2^ | df | F | p-value | | Partial η^2^ | df | F | p-value | Partial η^2^ |
| Hippocampus | 1,275 | 0.513 | 0.474 | 0.002 | 1,238 | 0.002 | 0.968 | | 0.000 | 1,236 | 1.839 | 0.176 | 0.008 |
| Amygdala | 1,275 | 4.757 | 0.030 | 0.017 | 1,238 | 0.272 | 0.603 | | 0.001 | 1,236 | 21.386 | 0.000 | 0.083 |
| Thickness caudal ACC | 1,275 | 0.085 | 0.771 | 0.000 | 1,238 | 0.488 | 0.485 | | 0.002 | 1,236 | 7.538 | 0.007 | 0.031 |
| Thickness rostral ACC | 1,275 | 1.093 | 0.297 | 0.004 | 1,238 | 0.676 | 0.412 | | 0.003 | 1,236 | 5.341 | 0.022 | 0.022 |
| SA caudal ACC | 1,274 | 0.507 | 0.477 | 0.002 | 1,237 | 2.987 | 0.085 | | 0.012 | 1,235 | 1.259 | 0.263 | 0.005 |
| SA rostral ACC | 1,274 | 0.917 | 0.339 | 0.003 | 1,237 | 2.004 | 0.158 | | 0.008 | 1,235 | 0.226 | 0.635 | 0.001 |
|  | | | | | BDNF gene expression  N = 195 | | | | | Childhood maltreatment x expression interaction N = 195 | | | |
|  | | | | | df | F | | p-value | Partial η^2^ | df | F | p-value | Partial η^2^ |
| Hippocampus | | | | | 1,181 | 1.160 | | 0.283 | 0.006 | 1,179 | 0.848 | 0.358 | 0.005 |
| Amygdala | | | | | 1,181 | 3.723 | | 0.055 | 0.022 | 1,179 | 8.158 | 0.005 | 0.044 |
| Thickness caudal ACC | | | | | 1,181 | 0.153 | | 0.696 | 0.001 | 1,179 | 0.611 | 0.436 | 0.003 |
| Thickness rostral ACC | | | | | 1,181 | 0.037 | | 0.848 | 0.000 | 1,179 | 4.631 | 0.033 | 0.025 |
| SA caudal ACC | | | | | 1,180 | 0.362 | | 0.548 | 0.002 | 1,178 | 0.715 | 0.399 | 0.004 |
| SA rostral ACC | | | | | 1,180 | 0.421 | | 0.517 | 0.002 | 1,178 | 0.056 | 0.814 | 0.000 |
|  | | | | | BDNF protein levels  N = 282 | | | | | Childhood maltreatment x protein interaction N = 282 | | | |
|  | | | | | df | F | | p-value | Partial η^2^ | df | F | p-value | Partial η^2^ |
| Hippocampus | | | | | 1,268 | 1.813 | | 0.179 | 0.007 | 1,266 | 0.900 | 0.344 | 0.003 |
| Amygdala | | | | | 1,268 | 0.073 | | 0.787 | 0.000 | 1,266 | 0.444 | 0.506 | 0.002 |
| Thickness caudal ACC | | | | | 1,268 | 0.019 | | 0.890 | 0.000 | 1,266 | 0.939 | 0.333 | 0.004 |
| Thickness rostral ACC | | | | | 1,268 | 0.081 | | 0.777 | 0.000 | 1,266 | 0.075 | 0.784 | 0.000 |
| SA caudal ACC | | | | | 1,267 | 0.198 | | 0.657 | 0.001 | 1,265 | 1.762 | 0.186 | 0.007 |
| SA rostral ACC | | | | | 1,267 | 0.731 | | 0.393 | 0.003 | 1,265 | 1.535 | 0.216 | 0.006 |

Table S2. Results of repeated measure ANOVA analyses after additional correction for smoking, alcohol use and use of selective serotonin reuptake inhibitors.

ACC: anterior cingulate cortex; Standardized beta values reported.

^a^ Additional correction for SSRI use, smoking and alcohol use

^b^ Additional correction for SSRI use, population structure, ancestry, smoking and alcohol use
